# Translation and reliability and validity of the Chinese version of Amyotrophic Lateral Sclerosis-Specific Quality of Life-Short Form

**DOI:** 10.1186/s41687-024-00738-4

**Published:** 2024-06-10

**Authors:** Zhijie Zhang, Xin He, Jialu Cui, Jing Wang, Baoxin Shi

**Affiliations:** 1 Hospice Care Research Center, Tianjin Medical University, 22 Qi Xiang Tai Road, Heping District, Tianjin 300070 China; 2 Tianjin Medical University Cancer Institute and Hospital, 24 Binshui Road, Hexi District, Tianjin 300060 China

**Keywords:** Amyotrophic lateral sclerosis, Quality of life, Scale, Chinese adaptation, Reliability, Validity

## Abstract

**Objective:**

To translate Amyotrophic Lateral Sclerosis-Specific Quality of Life-Short Form (ALSSQOL-SF) and test its reliability and validity, so that explore feasibility in Chinese mainland and make up the gap of specific tools for measuring quality of life of patients with ALS.

**Methods:**

This was a cross-sectional design. The Brislin translation model was used to translate ALSSQOL-SF, and the Chinese version of ALSSQOL-SF (C-ALSSQOL-SF) was revised through cultural adaptation and pre-test. The convenience sampling method was used to investigate 138 patients with ALS in Tianjin to test the reliability and validity of the C-ALSSQOL-SF.

**Results:**

The C-ALSSQOL-SF included 20 items, covering 6 dimensions: physical symptoms, bulbar function, negative emotion, interaction with people and the environment, religiosity and intimacy. The scale-level content validity index (S-CVI) of C-ALSSQOL-SF was 0.964, and the item-level content validity index (I-CVI) was between 0.857 to 1.000. The results of Confirmatory Factor Analysis (CFA) showed that CMIN/DF = 1.161, RMSEA = 0.034, GFI = 0.892, IFI = 0.976, TLI = 0.969, CFI = 0.975, and the 6-factor model fitted well. The scores of C-ALSSQOL-SF and WHOQOL-BREF were positively correlated (r = 0.745). The Cronbach’s α coefficient of the scale was 0.85, the Cronbach’s α coefficient of each dimension was between 0.59 to 0.86, and the split-half reliability was 0.78.

**Conclusion:**

The Chinese version of ALSSQOL-SF has good reliability and validity, and can be used as a tool to evaluate the quality of life of patients with ALS in Chinese mainland.

**Supplementary Information:**

The online version contains supplementary material available at 10.1186/s41687-024-00738-4.

## Introduction

Amyotrophic lateral sclerosis (ALS) involves the upper and lower motor neurons of central nervous system degenerative disease [1], with the progressive development of skeletal muscle weakness and atrophy, muscle tremor, bulbar paralysis and pyramidal tract as the main performance. Some patients with ALS may be accompanied by frontotemporal lobe involvement, such as different degrees of cognitive and/or behavioral impairment [2]. It is a rare neurological disease worldwide. In a survey of 727,718 people in seven provinces of Chinese mainland, the prevalence was 1.24/100,000 [3]; the annual incidence and prevalence of ALS reported in Hong Kong, China were 0.60/100,000 and 3.04/100,000, respectively [4]; and in Taiwan, China, the rates were 0.51/100,000 and 1.97/100,000, respectively [5]. In Europe, the prevalence of ALS was 2.2/100,000 [6], and in the United States, the prevalence in 2015 was 5.2/100,000, similar to that in 2014 (5.0/100,000) [7].

The loss of motor neurons is irreversible and the pathogenetic mechanisms of ALS are still not fully clear. There is no definitive cure for ALS, although Riluzole increases patient survival by three months [8]. Patients with ALS are faced with such problems as short survival, poor prognosis and reduced quality of life (QOL). The average survival time of most patients is usually 3–5 years, and most of them eventually die due to respiratory failure caused by respiratory muscle involvement [9–11]. The goal of treatment and nursing is to control symptoms, delay progression, and improve QOL. Medical staff should provide supportive care to patients, and reduce disease burden. Measuring and evaluating the QOL of patients with ALS can help medical staff find ways and methods to improve their QOL from a positive perspective, provide references for clinical improvement of their QOL, and better meet the needs of patients and their families.

At present, there are few types of research related to the QOL of patients with ALS in Chinese mainland, and there is a lack of multi-center and large-sample studies. Existing tools such as SF-36, WHOQOL-100, and WHOQOL-BREF can be used to measure patients with various diseases, with low sensitivity and specificity. There is a lack of clinical measurement tools specifically designed to measure QOL for patients with ALS. Amyotrophic Lateral Sclerosis-Specific Quality of Life Instrument (ALSSQOL) [12] and Amyotrophic Lateral Sclerosis-Specific Quality of Life-Short Form (ALSSQOL-SF) [13] are mature tools for evaluating the QOL of patients with ALS. However, compared with ALSSQOL, ALSSQOL-SF has fewer items and is more convenient to operate, which can be more effectively applied to patients. To provide an effective tool for measuring the QOL of patients with ALS in Chinese mainland, this study conducted translation, cultural adaptation, reliability and validity tests of the C-ALSSQOL-SF.

## Methods

### Study design

This was a cross-sectional design to translate ALSSQOL-SF and test its reliability and validity in China mainland.

### Translation, cultural adaptation and pre-test

After contacting Professor Zachary Simmons, we obtained his permission to use the scale. The C-ALSSQOL-SF was translated according to the Brislin translation mode [14]. The specific steps were as follows: (1) Forward translation: Two Chinese native speakers with a high level of English in nursing master’s degree independently translated the original English scale into Chinese. Then, the researchers discussed with them to form C-ALSSQOL-SF version 1. (2) Backward translation: The C-ALSSQOL-SF version 1 was independently translated into English by two university teachers who had not been seen to the original scale and were native English speakers and familiar with Chinese to get the backward translated version. (3) Cultural adaptation: Seven experts in neurology, nursing, and psychology were invited to form a cultural adaptation team to evaluate each dimension and item of the C-ALSSQOL-SF version 1, and to evaluate whether the contents of each dimension and item were clear and easy to understand, suitable for Chinese cultural background, language expression, and content relevance, then put forward suggestions for modification. The C-ALSSQOL-SF version 2 based on the opinions of experts was produced. There were 15 ALS patients recruited for pre-test with the C-ALSSQOL-SF version 2. Recorded the problems encountered by the patients in the process of responding, and asked them whether the expression of each item of the scale was easy to understand and their suggestions after completing the scale, and then adjustments were made. Finally, the formal Chinese version of ALSSQOL-SF was prepared.

In the cultural adaptation stage, after 2 rounds of consultation, according to the experts’ opinions, item 5 “My strength is affected” was changed to “My strength and activity are affected”, and item 18 “I am able to share my emotional condition with others” was changed to “I am able to talk about my emotion with others”. During the pre-test stage, the participants completed the responses within 5–7 min, and they indicated that the content of the scale was clear and easy to understand, so the content of the scale was not modified to form the final C-ALSSQOL-SF (Appendix File 1).

### Participants

Patients with ALS were recruited from Tianjin Grade III hospitals between December 2022 and June 2023 as the study subjects. Patients were eligible for the study had to meet the following criteria: (i) ≥18 years old; (ii) diagnosed according to the El Escorial revised criteria [15]; (iii) being able to understand Chinese; (iv) volunteering to participate and sign written informed consent. Exclusion criteria included: (i) previously diagnosed severe diseases (such as malignant tumors; lung, liver, and kidney damage); (ii) had a serious mental illness; (iii) had cognitive dysfunction. The sample size was 5–10 times the number of items on the scale. The ALSSQOL-SF has a total of 20 items. Considering the 20% shedding rate, the sample size was at least 120 ALS patients.

### Measurements

#### General demographic and clinical information

The information included age, gender, educational level, occupational status, marital status, religiosity, medical insurance, age of onset, location of onset, and duration of disease.

#### ALSSQOL-SF

In 2006, Simmons et al. [12] developed ALSSQOL for evaluating the QOL of ALS patients, ALSSQOL-SF [13] was further developed on the basis of its revised version for more convenient and efficient measurement of QOL in clinical applications in 2018. The English version of ALSSQOL-SF consisted of 20 items, covering 6 dimensions: physical symptoms, bulbar function, negative emotion, interaction with people and the environment, religiosity, and intimacy. The scale used the Likert scale, with options ranging from 0 for “strongly disagree” to 10 for “strongly agree”. Items 1–7, 10, 14 and 15 were reverse scoring items. An average score for each dimension was calculated, and the higher the score, the better the QOL. The Cronbach’s α coefficient of each dimension was 0.70, 0.81, 0.86, 0.80, 0.89, and 0.82, and the internal consistency was good. At present, this scale has been used in Brazil [16], Australia [17], and Italy [18] and so on.

#### WHOQOL-BREF

The WHOQOL-100 and WHOQOL-BREF scales have been developed by 15 centers around the world under the guidance of the World Health Organization and are now available in more than 20 languages. The WHOQOL-BREF is a good cross-cultural instrument for measuring the QOL around the world and has been widely used among ALS patients in previous studies [13, 16, 19]. A study has shown that the WHOQOL-BREF is valid for use in patients with ALS/MND [20]. Furthermore, considering the endurance and time problems of ALS patients, the WHOQOL-BREF is more suitable as the criterion-related scale to complete the survey task more efficiently. It [21] contained a total of 26 items in 4 fields: physical health, psychological, social relationships, and environment, including 2 independent items reflecting the overall health status: Question 1 asked about the overall subjective feelings about the quality of one’s life, Question 2 asked about the overall subjective feelings about one’s health status. The higher the score, the better the QOL. The WHOQOL-BREF is widely used in various fields in China with good reliability and validity.

### Statistical analysis

SPSS and AMOS were used for data analysis. Quantitative data that conform to normal distribution were represented by (*x *± *s*). The reliability and validity of the scale were tested by item analysis, content validity, construct validity, criterion-related validity, internal consistency reliability, and split-half reliability. *P*-values < 0.05 were considered statistically significant.

The item analysis was analyzed by the critical ratio (CR) test and correlation coefficient method. (1) The CR test was used to test the discrimination of each item. The C-ALSSQOL-SF scores of 138 ALS patients were ranked from high to low. The highest 27% of the total scores were classified as high group, and the lowest 27% were classified as low group. Independent-Samples t-test was performed on the two groups of data. The CR < 3 indicates that the discrimination of each item is poor. (2) The homogeneity of each item with the overall scale can be reflected by the correlation coefficient between the score of each item and the total score of the scale, and a correlation coefficient of r < 0.4 indicates a low correlation, the item should be considered for deletion.

The validity was tested by content validity, construct validity, and criterion-related validity. (1) The content validity was evaluated by the expert review method and expressed as content validity index (CVI). Seven experts from the fields of neurology, nursing, and psychology were invited to review the scale, and all experts had at least 10 years of working experience in their field. The correlation between each item of the C-ALSSQOL-SF and the corresponding dimension was evaluated using a 4-point Likert scale (1 = very irrelevant, 2 = irrelevant, 3 = quite relevant, and 4 = very relevant). Then, scale-level CVI (S-CVI) and item-level CVI (I-CVI) were calculated. The content validity of the scale can reflect the measured content well when I-CVI is above 0.78 and S-CVI is above 0.80 [22]. (2) The structural equation model was carried out using AMOS 26 software, and the construct validity of C-ALSSQOL-SF was tested using Conformation Factor Analysis (CFA). (3) The criterion-related validity was measured using the Pearson correlation analysis method by calculating the correlation between scores of C-ALSSQOL-SF and WHOQOL-BREF. Correlation coefficients values ≤ 0.19 were considered “very low”, values between 0.20 and 0.29 were considered “low”, values between 0.30 and 0.49 were considered “moderate”, and those between 0.50 and 0.69 were considered “high”. Correlation coefficients ≥ 0.70 were considered “very high” [23].

The reliability was tested by internal consistency reliability and split-half reliability. It is generally believed that the Cronbach’s α coefficient of the scale is not less than 0.8, and the Cronbach’s α coefficient of each dimension is not less than 0.6, indicating that the scale has good internal consistency [24]. The split-half reliability was obtained by dividing the C-ALSSQOL-SF into two groups according to the number of items, and the Spearman-Brown coefficient of the scores of the two parts was calculated, the split-half reliability was required to be above 0.7 [25].

## Results

### Participant demographics and characteristics

A total of 145 questionnaires were distributed and 138 participants completed the investigation, 4 participants did not have enough time to complete the investigation, 2 participants did not have the physical strength to complete the investigation, and one participant ultimately refused to complete the investigation. A total of 138 ALS patients were included in this study, including 80 males (58%) and 58 females (42%), with mean age of (61.86 ± 9.46) years, and the age of onset ranged from 38 to 83 years, with mean age of onset of (60.49 ± 9.52) years. Additional information was shown in Table 1.

**Table 1:** Characteristics of 138 ALS patients (N = 138)

Variables		N (%)
Gender	Males	80 (58.0)
	Females	58 (42.0)
Education level	Junior high school and below	71 (51.4)
	High school	42 (30.5)
	Three-year college	15 (10.9)
	University and above	10 (7.2)
Occupational status	Employment	18 (13.0)
	Retirees	33 (24.0)
	No fixed occupation	45 (32.6)
	Others	42(30.4)
Marital status	Unmarried	1 (0.7)
	Married	126 (91.3)
	Divorced	3 (2.2)
	Widowed	8 (5.8)
Religiosity	Yes	7 (5.1)
	No	131 (94.9)
Medical insurance	Yes	129 (93.5)
	No	9 (6.5)
Location of onset	Bulbar	30 (21.7)
	Limbs	97 (70.3)
	Others	11 (8.0)
Duration of disease	1 year blow	53 (38.4)
	1~2 years	46 (33.3)
	2 years above~3 years	23 (16.7)
	3 years above	16 (11.6)

### Item analysis

(1) The results showed that the CR values of each item ranged from 4.595 to 10.115 (all *P* < 0.01), and the discrimination of C-ALSSQOL-SF was good. (2) All correlation coefficients between items and total scores ranged from 0.403 to 0.686 (*P *< 0.01), indicating that each item was highly correlated with total C-ALSSQOL-SF.

### Validity analysis

**Fig. 1:**
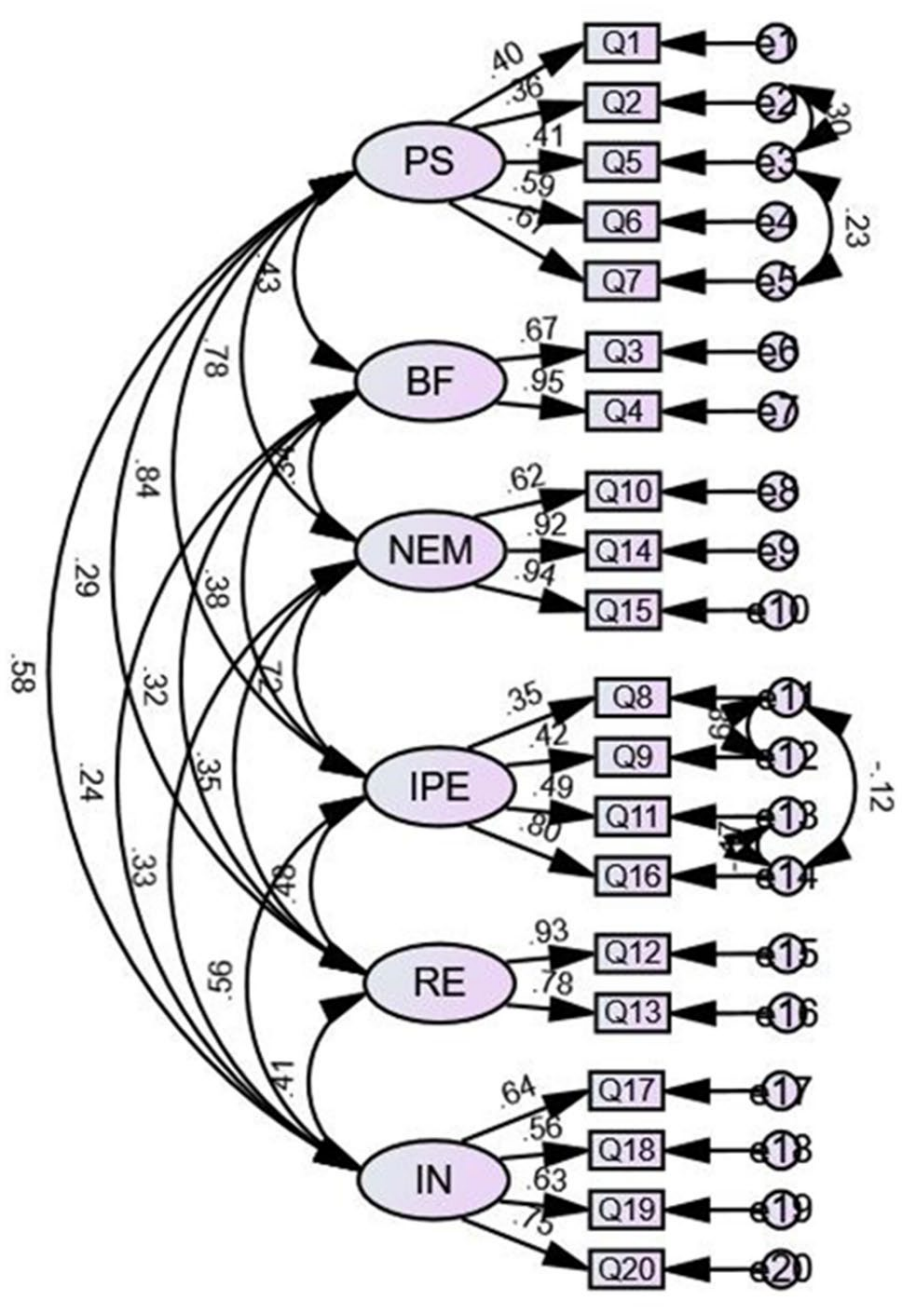
Modified model diagram

(1) In this study, the I-CVI value ranged from 0.857 to 1.000 and the S-CVI value was 0.964, which indicated that the content validity of C-ALSSQOL-SF was good. (2) The results of CFA showed that the initial model fitting effect was not ideal, and the model was modified according to Modification Indices (MI), the paths were e2 and e3, e13 and e14, e11 and e14, e3 and e5, e11 and e12. The results of the modified model showed that all fitting indexes had improved and basically met the reference standard (CMIN/DF = 1.161, RMSEA = 0.034, GFI = 0.892, IFI = 0.976, TLI = 0.969, CFI = 0.975). The modified model diagram and the twice fitting indexes were shown in Fig. 1 and Table 2. (3) The results of Pearson correlation analysis showed that scores of C-ALSSQOL-SF and WHOQOL-BREF were significantly very high correlation (r = 0.745, *P *< 0.01).

**Table 2:** Model fit test

Index	Reference standard	Initial model measurements	Modified model measurements
CMIN/DF	<3	1.606	1.161
RMSEA	<0.08	0.067	0.034
GFI	>0.9	0.860	0.892
IFI	>0.9	0.907	0.976
TLI	>0.9	0.882	0.969
CFI	>0.9	0.903	0.975

### Reliability analysis

(1) The Cronbach’s α coefficient of C-ALSSQOL-SF was 0.85, and the Cronbach’s α coefficient of each dimension ranged from 0.59 to 0.86. The detailed results and comparison with the original scale were shown in Table 3. (2) The results showed that the split-half reliability of C-ALSSQOL-SF was 0.78, and the correlation coefficient of the two groups was 0.635.

**Table 3:** Comparison of Cronbach’s α coefficient

Dimensions	Items	ALSSQOL-SF Cronbach’s α coefficient	C-ALSSQOL-SF Cronbach’s α coefficient
Physical symptoms	1, 2, 5, 6, 7	0.70	0.59
Bulbar function	3, 4	0.81	0.76
Negative emotion	10, 14, 15	0.86	0.86
Interaction with people and the environment	8, 9, 11, 16	0.80	0.68
Religiosity	12, 13	0.89	0.84
Intimacy	17, 18, 19, 20	0.82	0.73
The scale	1~20	–	0.85

## Discussion

In China, fewer studies have been conducted on the QOL of ALS patien, and most of them have generally utilized universal QOL scales that were not specific to ALS patients and could not comprehensively and accurately reflect the real situation of the QOL of ALS patients. Effective measurement of QOL of ALS patients could help medical staff take targeted interventions to reduce the burden of symptoms and improve the QOL of patients. Based on the current situation, we need a reliable tool to assess the QOL of ALS patients.

Validity refers to the degree to which the measurement results of the scale can accurately reflect the measured content. In this study, the validity of C-ALSSQOL-SF was tested by content validity, construct validity, and criterion-related validity. Firstly, the expert review method was used to examine content validity, the S-CVI > 0.9 and I-CVI > 0.8 of C-ALSSQOL-SF in this study, indicating good discriminability and adaptability of each item and good content validity of the scale. Secondly, the original dimension of the original scale has been divided, so this study used CFA to test the construct validity of the original 6-factor model. The results showed that the fitting results of the initial 6-factor model were not ideal (GFI = 0.860 and TLI = 0.882 were not ideal). Therefore, the original model was modified according to the MI and the fitting indexes of the modified model basically met the reference standards, it could pass the model fit test, indicating that the construct validity of C-ALSSQOL-SF was acceptable. Thirdly, we compared the correlation between scores of C-ALSSQOL-SF and WHOQOL-BREF, the result showed that there was a significant correlation, indicating good criterion-related validity. In conclusion, the Chinese version of ALSSQOL-SF has good validity in the application of ALS patients, which is consistent with results of studies of other versions. The CFA results of the original scale showed that the model fit was acceptable: χ2 (165) = 349.92; *P* < 0.001; CFI = 0.857; RMSEA = 0.083; and SRMR = 0.10, and the ALSSQOL-SF and its subscales had construct, convergent, and divergent validity. The Portuguese version of ALSSQOL-SF [16] also passed convergent validity and divergent validity tests using correlation analysis.

Reliability represents the consistency and stability of the measurement results of the scale. In this study, the Cronbach’s α coefficient of the C-ALSSQOL-SF was 0.85, indicating the overall scale had excellent internal consistency. The Cronbach’s α coefficient of all dimensions ranged from 0.59 to 0.86, which were similar to the original scale (0.70–0.89) [13] and the split-half reliability was 0.78. The dimension with the highest Cronbach’s α coefficient was negative emotion, with a value of 0.86, but the original scale had the highest score for the religiosity dimension, with a value of 0.89, and the lowest dimension was physical symptoms, with a value of 0.59, which was consistent with the result of the original scale, which had a value of 0.70. The Portuguese version has not been tested for reliability, and the reliability of the scale needs to be tested in more cultural contexts in the future. In this study, except for the dimension of physical symptoms, the Cronbach’s α coefficient of other dimensions were all more than 0.6, but the Cronbach’s α coefficient of physical symptoms dimension was 0.59, which was very close to the standard, so we considered the internal consistency of this dimension to be acceptable. The low Cronbach’s α coefficient of this dimension in this study was mainly due to the following reasons. First, the Cronbach’s α coefficient of this dimension in the original scale was 0.70, the lowest among the 6 dimensions, which was consistent with the results of this study. Second, item 1 of physical symptoms, “pain” was a non-motor symptom, not a specific symptom of ALS, and the pain may also be related to the course of patients’ disease. There was a large difference between different patients, which may lead to decreased internal consistency in this dimension. However, after deleting this item in the reliability test, the Cronbach’s α coefficient of the scale did not improve, so it was retained. Finally, it may be related to the small sample size of this study. As ALS is a rare disease with a low prevalence rate and easy delay in diagnosis, sample collection had some difficulties, and the sample size only met the minimum requirements. In addition, internal consistency reliability was not the only criterion to judge the reliability of the scale, and other relevant indexes and application effects should be comprehensively considered in actual research [26]. Therefore, through the reliability test, the C-ALSSQOL-SF has acceptable reliability.

Improving quality of life is an important part of long-term care for ALS patients, and targeted tools are needed to complete the measurement and provide a basis for developing interventions. This scale is a specific scale for ALS patients, including the inquiry of disease-related symptoms and psychosocial problems, which can comprehensively evaluate the QOL patients, and has high application value. This study strictly followed the Brislin translation model, and the C-ALSSQOL-SF was formed after repeated cultural adaptation and pre-test, which showed good reliability and validity in the application of ALS patients in Chinese mainland, with significant correlation between each item of the scale and the total score of the scale, and the correlation coefficient was > 0.4. All items were retained. The number of items is moderate, the content is easy to understand, and it is convenient to complete, and study subjects can complete the answer within 5 to 7 minutes, which has high clinical applicability and operability. Therefore, the C-ALSSQOL-SF can be used as a reliable tool to evaluate the QOL of ALS patients in Chinese mainland.

### Limitations

There are still several limitations in this study. First, the selected subjects were concentrated in Tianjin, and the sample representation was insufficient. Second, due to the characteristics of the patient population, the sample size was small. Third, our study was a cross-sectional study, so it is necessary to increase the sample size, expand the research scope, and conduct longitudinal research in the future to further examine the reliability and validity of C-ALSSQOL-SF.

## Conclusion

The Chinese version of ALSSQOL-SF contains 6 dimensions and 20 items. The six dimensions were physical symptoms (items 1, 2, 5, 6, 7), bulbar function (items 3, 4), negative emotion (items 10, 14, 15), interaction with people and the environment (items 8, 9, 11, 16), religiosity (items 12, 13), and intimacy (items 17, 18, 19, 20), with good reliability and validity. It can be used to investigate the quality of life of patients with ALS, help medical staff to evaluate the status of ALS patients in time, and provide targeted interventions.

### Electronic supplementary material

Below is the link to the electronic supplementary material.

Supplementary Material 1

## Data Availability

The datasets generated and/or analysed during the current study are not publicly available due the data still needs to be used for other research but are available from the corresponding author on reasonable request.

## Abbreviations

ALS: Amyotrophic lateral sclerosis
ALSSQOL: Amyotrophic lateral sclerosis-specific quality of life instrument
ALSSQOL-SF: Amyotrophic lateral sclerosis-specific quality of life-short form
C-ALSSQOL-S: Chinese version of ALSSQOL-SF
QOL: Quality of life
CR: Critical ratio
CVI: Content validity index
S-CVI: Scale-level CVI
I-CVI: Item-level CVI
CFA: Confirmatory factor analysis
MI: Modification indices
