# Supplementary material for: Translation and reliability and validity of the Chinese version of Amyotrophic Lateral Sclerosis-Specific Quality of Life-Short Form
Source: J Patient Rep Outcomes. 2024 Jun 10;8:57. doi: 10.1186/s41687-024-00738-4 (PMC11164839; doi:10.1186/s41687-024-00738-4)
Supplement: Supplementary file 1 — Supplementary Material 1 [file 41687_2024_738_MOESM1_ESM.doc]

Additional File 1 The Chinese version of ALSSQOL-SF (C-ALSSQOL-SF)

|  | **非常不同意** |  |  |  |  |  |  |  |  |  | **非常**  **同意** |
| --- | --- | --- | --- | --- | --- | --- | --- | --- | --- | --- | --- |
| 1.我感到疼痛 | 0 | 1 | 2 | 3 | 4 | 5 | 6 | 7 | 8 | 9 | 10 |
| 2.我感到疲劳 | 0 | 1 | 2 | 3 | 4 | 5 | 6 | 7 | 8 | 9 | 10 |
| 3.我的口水过多 | 0 | 1 | 2 | 3 | 4 | 5 | 6 | 7 | 8 | 9 | 10 |
| 4.我的讲话能力受到影响 | 0 | 1 | 2 | 3 | 4 | 5 | 6 | 7 | 8 | 9 | 10 |
| 5.我的力气和活动能力受到影响 | 0 | 1 | 2 | 3 | 4 | 5 | 6 | 7 | 8 | 9 | 10 |
| 6.我的睡眠受到影响 | 0 | 1 | 2 | 3 | 4 | 5 | 6 | 7 | 8 | 9 | 10 |
| 7.我觉得身体状况很糟糕 | 0 | 1 | 2 | 3 | 4 | 5 | 6 | 7 | 8 | 9 | 10 |
| 8.我的需求能够得到关注和回应 | 0 | 1 | 2 | 3 | 4 | 5 | 6 | 7 | 8 | 9 | 10 |
| 9.我感觉能够得到支持 | 0 | 1 | 2 | 3 | 4 | 5 | 6 | 7 | 8 | 9 | 10 |
| 10.我一直很沮丧 | 0 | 1 | 2 | 3 | 4 | 5 | 6 | 7 | 8 | 9 | 10 |
| 11.我很满意与亲人的关系 | 0 | 1 | 2 | 3 | 4 | 5 | 6 | 7 | 8 | 9 | 10 |
| 12.信仰能够给我力量/安慰 | 0 | 1 | 2 | 3 | 4 | 5 | 6 | 7 | 8 | 9 | 10 |
| 13.我坚信自己的信仰 | 0 | 1 | 2 | 3 | 4 | 5 | 6 | 7 | 8 | 9 | 10 |
| 14.我感到绝望 | 0 | 1 | 2 | 3 | 4 | 5 | 6 | 7 | 8 | 9 | 10 |
| 15.我感到悲伤 | 0 | 1 | 2 | 3 | 4 | 5 | 6 | 7 | 8 | 9 | 10 |
| 16.我能够感受到周围事物的美好 | 0 | 1 | 2 | 3 | 4 | 5 | 6 | 7 | 8 | 9 | 10 |
| 17.我很渴望亲密感情 | 0 | 1 | 2 | 3 | 4 | 5 | 6 | 7 | 8 | 9 | 10 |
| 18.我能够与他人诉说自己的情感 | 0 | 1 | 2 | 3 | 4 | 5 | 6 | 7 | 8 | 9 | 10 |
| 19.我很渴望亲密接触 | 0 | 1 | 2 | 3 | 4 | 5 | 6 | 7 | 8 | 9 | 10 |
| 20.我和他人有亲密接触 | 0 | 1 | 2 | 3 | 4 | 5 | 6 | 7 | 8 | 9 | 10 |
